# An Interoperability Platform Enabling Reuse of Electronic Health Records for Signal Verification Studies

**DOI:** 10.1155/2016/6741418

**Published:** 2016-03-31

**Authors:** Mustafa Yuksel, Suat Gonul, Gokce Banu Laleci Erturkmen, Ali Anil Sinaci, Paolo Invernizzi, Sara Facchinetti, Andrea Migliavacca, Tomas Bergvall, Kristof Depraetere, Jos De Roo

**Affiliations:** ^1^SRDC Software Research & Development and Consultancy Ltd., 06800 Ankara, Turkey; ^2^Department of Computer Engineering, Middle East Technical University, 06800 Ankara, Turkey; ^3^Lombardia Informatica S.p.A., Via Torquato Taramelli, 26 20124 Milano, Italy; ^4^WHO Collaborating Centre for International Drug Monitoring, Uppsala Monitoring Centre (UMC), 753 20 Uppsala, Sweden; ^5^Advanced Clinical Applications Research Group, Agfa HealthCare, 9000 Gent, Belgium

## Abstract

Depending mostly on voluntarily sent spontaneous reports, pharmacovigilance studies are hampered by low quantity and quality of patient data. Our objective is to improve postmarket safety studies by enabling safety analysts to seamlessly access a wide range of EHR sources for collecting deidentified medical data sets of selected patient populations and tracing the reported incidents back to original EHRs. We have developed an ontological framework where EHR sources and target clinical research systems can continue using their own local data models, interfaces, and terminology systems, while structural interoperability and Semantic Interoperability are handled through rule-based reasoning on formal representations of different models and terminology systems maintained in the SALUS Semantic Resource Set. SALUS Common Information Model at the core of this set acts as the common mediator. We demonstrate the capabilities of our framework through one of the SALUS safety analysis tools, namely, the Case Series Characterization Tool, which have been deployed on top of regional EHR Data Warehouse of the Lombardy Region containing about 1 billion records from 16 million patients and validated by several pharmacovigilance researchers with real-life cases. The results confirm significant improvements in signal detection and evaluation compared to traditional methods with the missing background information.

## 1. Introduction

All medicinal products are subject to strict testing and assessment of their quality, efficacy, and safety before being authorized. While premarket safety analysis through clinical trials remains vital, there is considerable attention towards improving the reporting and collection of postmarket data to enhance patient safety. After authorization, all medicinal products continue to be observed through pharmacovigilance studies to monitor their safety profiles. Currently, pharmacovigilance activities are mainly based on signal detection studies run on voluntarily sent spontaneous reports. Although spontaneous reporting remains a cornerstone of pharmacovigilance in the regulator environment and is indispensable for signal detection, due to examples of drug withdrawals [1] stemming from uncommon adverse events after millions of patients were exposed, the need for a more effective and proactive surveillance is reinforced.

The current postmarket drug surveillance process has several bottlenecks, with the first one being underreporting [2, 3]; it has been estimated that only about 5% of harmful Adverse Drug Events (ADEs) (Abbreviations are provided at the end of the paper) are being reported through spontaneous reporting [4, 5]. Secondly, the quality of the data collected through spontaneous reporting is low [6], and finally spontaneous reports only report adverse incidents, while the information related to other patients who used the drug but not experienced adverse events, that is, the denominator data, is not retrievable [7].

For these reasons, there is a clear need for complementary pharmacovigilance activities. Relative to Individual Case Safety Reports (ICSRs), Electronic Health Records (EHRs) cover extended parts of the underlying medical histories, include more complete information on potential risk factors, and are not restricted to patients who have experienced a suspected ADE [8]. Hence, there is great potential in accessing EHRs for tracing safety reports back to medical summaries of patients and also secondary use of EHRs for complementary pharmacoepidemiology studies for clinical signal evaluation and validation. For example, Uppsala Monitoring Centre (UMC) on behalf of the WHO International Programme for International Drug Monitoring analyses the WHO global ICSR database, VigiBase, for potential signals [9, 10]. The objective is to characterize the reported cases in comparison with a selected background population for checking whether there are other explanations more likely to cause the reported adverse event than the exposure to drug of interest. Yet, the data sets used for such studies are limited both in quantity and also in extent of medical information covered and geographical spread. Accessing a wide range of EHR sources seamlessly to collect the background information of any selected patient population, more importantly tracing the reported incidents back to original EHRs, can provide major improvements for such clinical validation studies, as we demonstrate in this paper.

This paper presents the interoperability framework developed in the SALUS (Scalable, Standard based Interoperability Framework for Sustainable Proactive Post Market Safety Studies) project [11], which enables effective integration and utilization of EHR data to reinforce postmarket safety activities. The interoperability architecture addresses both structural interoperability and Semantic Interoperability through an ontological framework. The objective is to enable safety analysts to seamlessly access EHR data from heterogeneous healthcare systems. In this way, they will be able to trace ICSRs back to EHRs and collect deidentified medical data sets of selected populations to run complementary safety analysis studies.

Postmarket safety studies cover a wide area where various analyses can be done by following different approaches. Therefore, as one of the first activities in the SALUS project, we have identified the concrete pilot application scenarios to be implemented. We have agreed on six pilot application scenarios, four of which are specific safety analysis methods for different purposes, while the remaining two are focused on semiautomatic ADE notification and reporting.

Our work in this paper first provides the underlying Semantic Interoperability Framework that is used commonly in all six pilot application scenarios. Furthermore, this paper focuses on the implementation and validation of one of the safety analysis methods, namely, the* case series characterization scenario* that aims at adding meat to the bones of the potential signals by characterizing the cases (i.e., foreground population) and contrasting them to a background population. The underlying interoperability framework and all Web-based SALUS tools including the Case Series Characterization Tool (CSCT) have been deployed in Lombardy Region in Italy and Technical University of Dresden Hospital in Germany. This paper focuses on the deployment and validation activities on top of the huge regional EHR Data Warehouse of Lombardy containing about 1 billion records from 16 million patients. This CSCT deployment has been validated by pharmacovigilance researchers from both UMC and Lombardy Regional Pharmacovigilance Centre and presented to Italian National Pharmacovigilance Agency (AIFA).

## 2. Background and Significance

Recently, a number of investigators have examined potential use cases for secondary use of EHR data in clinical research and patient safety contexts including eligibility determination, clinical trial data collection, adverse event reporting, and conduction of epidemiological studies [12–21]. Murphy et al. [22] describe the potential of using routinely collected clinical data for conducting retrospective observational studies.

Although reuse of EHRs for safety studies has a great potential, a major barrier is that information systems in patient care and clinical research domains are not interoperable with each other. This is due to the fact that different reference information models (as models of use) such as HL7 RIM [23], ISO/CEN 13606 Reference Model [24], CDISC ODM [25], BRIDG DAM [26] and different standard terminology systems (as models of meaning) such as ICD-9, ICD-10, SNOMED-CT, Medical Dictionary for Regulatory Activities (MedDRA), and CDISC Terminology are used in care and research domains. Hence, although the required information for the safety analysis studies is available in EHR systems, it is not readily available in a structurally and semantically interoperable manner.

There are several efforts for addressing this interoperability challenge. Some approaches like OMOP [27], Mini-Sentinel [28], EU-ADR [29, 30], and SHRINE [31] define their own Common Information Models and the corresponding data repository schemas and request participating data sources to fill in a central data repository by converting data in their native model to the corresponding fixed schema. SHARPn is a similar project [32], and it supports some standards such as HL7 v2.x messages and Clinical Document Architecture (CDA) as well, with the extended capability of natural language processing. Still, as in the case of the above-mentioned approaches, data of a fixed population is transferred manually in advance and hence dynamic eligibility criteria execution on top of the actual data sources is not supported.

Integrating the Healthcare Enterprise (IHE) profiles [33] selects and assumes conformance to a well-defined interface standard to communicate with EHR systems, like HL7/ASTM Continuity of Care Document (CCD) [34] to share medical summaries of patients. IHE Drug Safety Content [35] and Clinical Research Data Capture [36] profiles address the structural interoperability of care and research domains by proposing XSLT mapping between different information models used in clinical care (HL7/ASTM CCD) and clinical research domains (CDASH [37] annotated CDISC ODM and ICH E2B(R2) [38]).

Several other efforts like Artemis [39], ACGT [40], and DebugIT [41, 42] follow a mediation approach, where the local models are formalized as ontologies and mediated to one another based on a global model. Lezcano et al. [43] demonstrate reasoning on clinical knowledge through a single content model, the semantic representation of ISO/CEN 13606 archetypes.

When it comes to addressing Semantic Interoperability mismatches due to the use of different terminology systems, in some efforts like epSOS [44], SHARPn, OMOP, and DebugIT, pivot terminology systems are mandated. Some other efforts handle this separately by calls to external terminology systems like UMLS [45] and LexEVS [46] (Artemis, iCARDEA [47], and TrialX [48, 49]).

We believe that addressing syntactic and Semantic Interoperability cannot be separated from each other, since the binding between models of use and models of meaning also has an impact on Semantic Interoperability [50, 51]. In this work, we propose an ontological framework where each local system can continue to use its own local models and terminology systems, while both structural mapping and terminology mapping are handled through rule-based reasoning on formal representations of reference models and terminology systems.

TRANSFoRm project also proposes a unified framework for representing structural and semantic models to address the interoperability problem [52], but through a terminology server, LexEVS. In our work, we demonstrate that representing all the knowledge through formal means, as ontologies, and establishing the necessary links again through ontological constructs give an enhanced capability of semantic mediation and terminology reasoning.

## 3. Materials and Methods

The aim of a case series characterization study is to evaluate the validity of a potential signal, that is, the effect of a specific drug on a specific event. In particular, the safety analysts working at UMC and national/regional pharmacovigilance bodies are trying to find answers to such problems.

- What differs between the patients having a* Myocardial Infarction (MI)* within 2 weeks of* Nifedipine* intake (foreground population) and all patients taking* Nifedipine* (background population)?
- What is the proportion of male patients in both foreground and background populations, where patients using* Ramipril* and having a* Pancreatitis* reaction within 120 days after prescription compose foreground population and all patients using* Ramipril* compose background population?
- A signal of* Pancreatitis* associated with the usage of* Amiodarone* has been communicated to the Pharmacovigilance Risk Assessment Committee. Scientific literature on this specific topic is rather limited. Experts timely need to analyse the foreground population (*Amiodarone* and* Pancreatitis* after drug intake) in comparison with the background population (patients treated with antiarrhythmics) to evaluate the associated risk and to have an insight of comorbidities and concomitant drugs.

Safety analysts need to access medical data sets of selected foreground and background populations from disparate EHR systems to be able to check whether there are other explanations more likely to cause a specific event (e.g.,* Pancreatitis*) than the exposure to the specific drug (e.g.,* Ramipril*).

**Figure 1:**
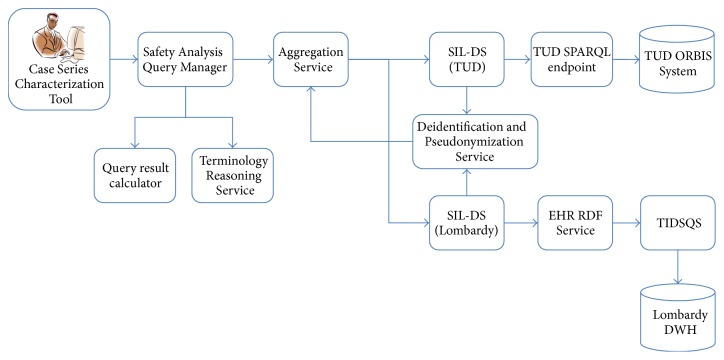
Components of the SALUS architecture involved in case series characterization implementation.

SALUS Framework enables the execution of such use case scenarios through a series of integrated semantic and technical interoperability components, as displayed in Figure 1. In brief, Safety Analysis Query Manager receives the query parameters from the Web-based query tool for safety analysts and forwards them to the Semantic Interoperability Layer-Data Services (SIL-DSs) through the Aggregation Service, which is responsible for the aggregation of data coming from different data sources. SIL-DSs and the supporting SALUS components deployed on top of the EHR data sources handle the structural and semantic conversion of query parameters and the returning eligible patient data. Conversion of codes among different clinical care and clinical research terminology systems is realized by the Terminology Reasoning Service. Privacy of data is ensured by the Deidentification and Pseudonymization Service.

In the upcoming sections, all these components displayed in Figure 1 are presented in detail by focusing on the addressed challenges. Further details about the SALUS Interoperability Framework can be found in [53].

### 3.1. Query Tool for Safety Analysts

**Figure 2:**
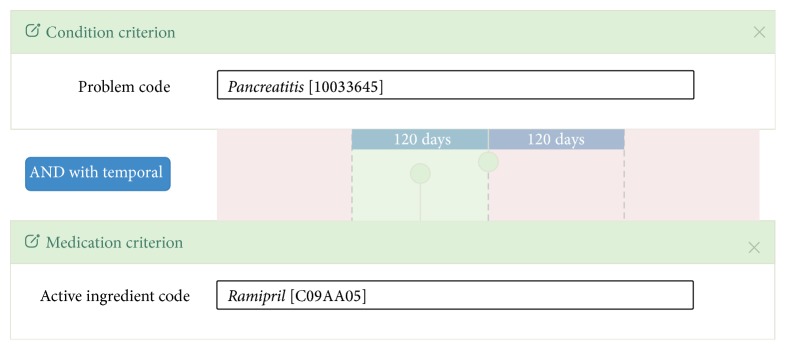
Eligibility criteria definition interface of the CSCT. In this example, the analyst defines* Pancreatitis* condition and* Ramipril* medication for the foreground population by also adding a temporal relation stating that the former shall occur within 120 days latter's occurrence.

SALUS Framework provides the Case Series Characterization Tool (CSCT) as a Web application, which enables the safety analyst to formally define the characteristics of foreground and background populations. It is possible to define eligibility criteria by expressing several different clinical statements, such as conditions, medications, lab results, and procedures, which are retrieved from a common model, SALUS Common Information Model (CIM). Such criteria are represented by selecting coded values from terminology systems; for example, the medical event of interest can be defined by selecting* Pancreatitis* MedDRA Preferred Term (PT) and medication of interest by selecting* Ramipril* from WHO-ATC (see Figure 2). The terminology systems to be used in these fields are configurable; for example, another analyst may prefer to use SNOMED-CT for defining problem codes. For enabling efficient type-ahead search functionality during code selection, the tool is integrated with a terminology server that indexes medical terminologies. It is also possible to define logical operators (e.g., AND, OR) and temporal constraints (e.g., within 120 days) among different criteria.

The tool also enables the safety analyst to configure the statistics to be calculated for grouping and stratifying data sets of the eligible populations, such as age, gender, and common conditions/medications before/after medication/event of interest. The coded data can be configured to be grouped under a preferred terminology system and level in the results, for example, MedDRA High Level Group Terms (HLGT), no matter which specific terminology system is used in the EHR sources. Finally, it is possible to define a number of coded risk factors to be specifically checked on both populations. These represent the possible confounding factors of the selected conditions in the eligibility criteria that need to be checked in the medical summaries of the eligible patients, such as diabetes and obesity.

The eligibility criteria need to be passed to disparate EHR sources, and the deidentified medical data sets should be retrieved for the eligible patients. After aggregation, these medical data sets need to be analysed to calculate the statistical information asked by the safety analyst. However, there are several challenges: (i) divergent data models are used to represent EHRs and (ii) several different terminology systems are used to code structured patient data. In our architecture, we address these problems by formalizing the local models of EHR sites and semantically aggregating them using a common model, which we call SALUS Common Information Model (CIM). SALUS CIM is linked with ontological representations of terminology systems; hence, before the statistics are calculated on the aggregated data represented in CIM, terminology reasoning is handled to address not only structural but also semantic mismatches between data sources and the requestor.

### 3.2. EHR Sources and Formalizing EHR Data

There are two EHR sources in SALUS.

- A Regional Health Data Warehouse (DWH) is maintained in Lombardy Region in Italy, which collects and extracts all data necessary for administrative and statistical purposes from almost all the public healthcare providers. It is operational since 2002, covering medical records of around 16 million patients. This huge DWH includes around 1 billion records including hospitalizations, ambulatory events, chronic conditions, drug prescriptions, allergies, vaccinations, and pregnancies. Its main advantage is providing longitudinal data from all public healthcare providers at the primary, secondary, and tertiary levels. Also, all data in the DWH is structured and coded. In SALUS, we are using a copy of the DWH for both eliminating unnecessary data (e.g., financial) and not affecting the regular operation of the system. This DWH has a monthly update mechanism according to the data flow time process of the regional DWH. All the information present in the regional DWH is structured and coded.
- The second source is the AGFA ORBIS installation used as the EHR system at Technical University of Dresden (TUD) Hospital, which is the largest hospital structure with 21 clinics in Saxony, Germany. For use in SALUS, access to a live backup of the operational TST1 database is provided, which includes data of around 950 thousand patients with around 75 million records including 13 million diagnoses, 2 million medications, and 56 million lab results.

In SALUS, we follow a nondisruptive approach and collect EHR data in the local models used by the EHR systems. These can be based on interface standards as in the case of Lombardy DWH, which can provide medical data represented in CCD/Patient Care Coordination (PCC) templates [54] or proprietary formats like ORBIS relational data model as in the case of TUD. In both cases, in order to proceed with semantic mediation, the first thing that has to be done is formalizing the retrieved EHR data by representing them as Resource Description Framework (RDF) [55] entities in local ontologies corresponding to the local models, which we prefer to call “Content Entity Model.”

Before SALUS, Lombardy Regional Health Infrastructure was already able to produce and exchange patient summary documents complying with CCD/PCC templates within the scope of epSOS project [56]. Building on the results of epSOS, in SALUS, we would like to enable the collection of deidentified medical summaries represented in CCD/PCC templates for the use of clinical research studies through standard based transactions. For this purpose, we have extended the native IHE Query for Existing Data (QED) [57] transactions to support population based queries and to provide data of all eligible patients represented in CCD/PCC templates as usual [58]. Eligibility criteria are represented in HL7 Health Quality Measures Format (HQMF) [59] to express population based queries. In the SALUS architecture, Technical Interoperability Data Source Query Service (TIDSQS) implements the extended QED profile on top of Lombardy DWH.

**Figure 3:**
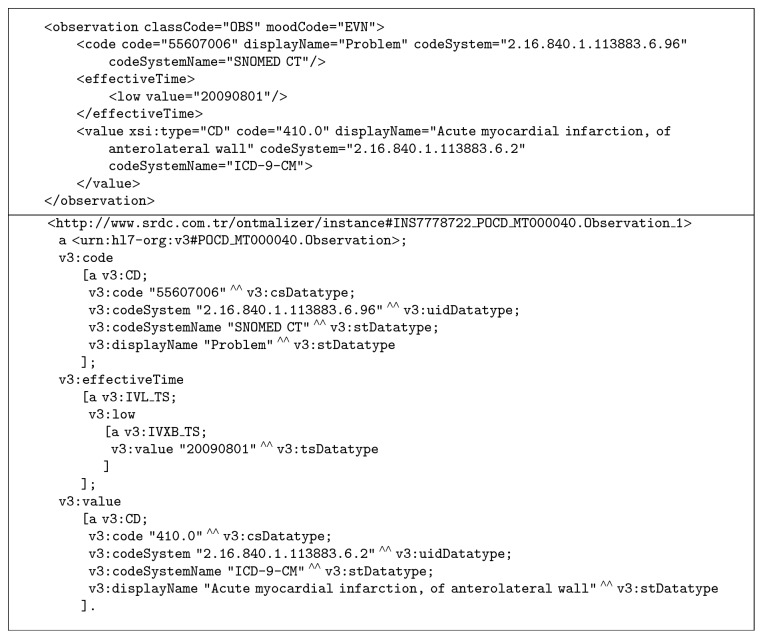
A simple HL7 CDA observation instance for* Acute myocardial infarction* in its native XML syntax and the corresponding RDFized (i.e., formalized) instance in N3 syntax.

The EHR RDF Service gets the data of the eligible patients from TIDSQS in native XML representation of the CCD/PCC templates, after which data formalization takes place. In order to perform comprehensive transformations of XML Schemas (XSD) and XML data to RDF automatically, we have implemented a tool named Ontmalizer [60]. Through this tool, the CCD/PCC template instances retrieved from TIDSQS complying with HL7 CDA Schema [61] are automatically RDFized by creating the corresponding ontology instances. The outcome is always a one-to-one correspondence of the input data but represented as RDF entities to foster further semantic processing. A simple HL7 CDA observation instance in its native XML syntax and its one-to-one RDFized correspondence in Notation 3 (N3) syntax [62] is provided in Figure 3.

A slightly different approach is followed on the TUD side. Instead of data exchange through some content standards, a SPARQL [63] endpoint is exposed directly on top of the TUD ORBIS System, which is able to retrieve data from the relational tables of ORBIS and return as RDF entities in the ORBIS Content Entity Model. In this case, EHR data formalization immediately takes place on top of the relational database.

### 3.3. SALUS Common Information Model (CIM)

**Figure 4:**
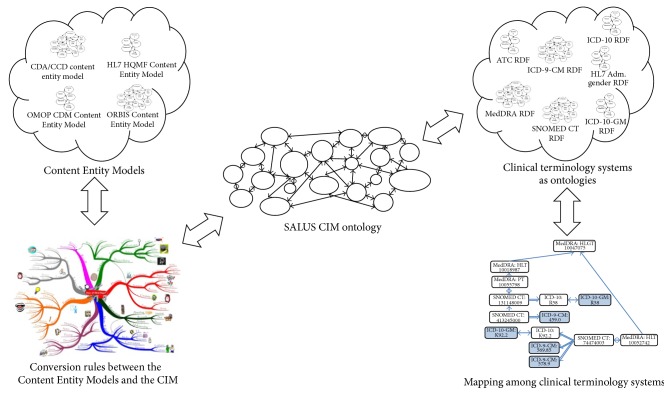
The Semantic Resource Set as the backbone enabling the SALUS Semantic Interoperability Framework.

SALUS Common Information Model (CIM) ontology forms the core of the SALUS Semantic Resource Set (see Figure 4), with the aim of preventing n-to-n mapping among varying content models of data sources and requestors.

During the requirements analysis phase, we have collected all the clinical data requirements of our pilot application scenarios; one among six is the case series characterization. Although the requirements of our pilot applications were our main driving point, we have analysed and taken into account content models from other standards and initiatives as well, to provide a common mediator that can interoperate with well-established state of the art. These include HL7/ASTM CCD and IHE PCC templates, HITSP C32/C83 components [64, 65], Consolidated CDA templates [66], Observational Medical Outcomes Partnership (OMOP) Common Data Model (CDM) [67], ICH E2B(R2), and ISO/CEN EN 13606 archetypes.

As a result, we have built a list of Common Data Elements (CDEs) that include elements to be present within a medical summary, such as patient demographics, encounter, condition (problem, diagnosis), allergy, family history, and healthcare provider, and their subelements [68]. After identifying the required CDEs, we first created the SALUS CIM as an XSD containing all the CDEs and the relationships among them. In addition to the CDEs, we have also used a simple yet satisfactory subset from ISO 21090 data types [69] including the most essential data types such as concept descriptor (CD), interval of timestamp (IVLTS), instance identifier (II), and physical quantity (PQ). As the next step, we transformed this XSD into the SALUS CIM ontology automatically by using Ontmalizer. Finally, we have done some manual updates on the RDF representation to appropriately reuse the existing ontologies and terminologies such as foaf, schema.org, and SNOMED-CT [70]. This strategy has been chosen to avoid creating from scratch the entities that are already defined by the existing resources and to favor the reuse of our entity models in the healthcare and EHR communities.

Composed of 211 CDEs, SALUS CIM ontology acts as a mediator among different content models. SALUS CIM ontology not only represents entities that can be presented within a medical summary, but also establishes a link with the terminology system ontologies that are used to code patient data.

SALUS CIM also covers the query model to express eligibility criteria for defining a population of interest. For this purpose, we mainly benefited from the query model of HL7 HQMF and created its semantic representation within the SALUS CIM ontology.

None of the above-mentioned existing models is satisfactory enough in terms of scope to meet the requirements of observational studies on its own. Therefore, we had to develop the SALUS CIM as a harmonization of several well-accepted content models used in the clinical care and observational study domains.

### 3.4. Conversion to SALUS CIM Instances

In our architecture, Semantic Interoperability Layer-Data Services (SIL-DSs) for Lombardy and TUD are responsible for converting the medical summaries of the eligible population represented in local ontologies, that is, CDA/CCD Content Entity Model instances received from EHR RDF Service and ORBIS Content Entity Model instances received from TUD SPARQL Endpoint to instances represented in SALUS CIM Ontology. In order to perform this operation, a set of conversion rules in Notation 3 (N3) [62] has been implemented in Euler Yap Engine (EYE), which is an open source and high performance reasoning engine maintained by AGFA [71]. We have 75 high level conversion rules for mapping CDA/CCD Content Entity Model to SALUS CIM. Our conversion approach is described in more detail in a similar previous work [72].

Content Entity Models and conversion rules are part of the SALUS Semantic Resource Set. Whenever a new content model is to be introduced in the SALUS architecture, it is necessary to define the conversion rules from the corresponding entity model (i.e., formalized) to the SALUS CIM Ontology as the common mediator. This is a one-time manual process. Although the CIM has become quite mature after several iterations, still it can be the case that it would not cover a new content model completely. In this case, the CIM is extended without disrupting the existing data elements so that it covers the new content model to be mapped while preserving the existing conversion rules.

### 3.5. Running Queries over Semantic Interoperability Framework

**Figure 5:**
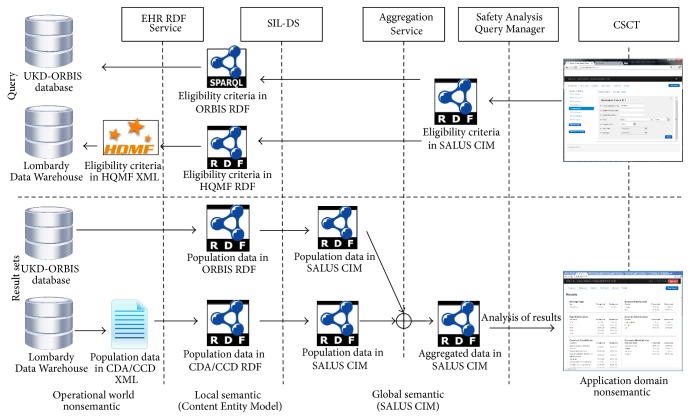
The complete transformation and mediation cycle of the eligibility query and the result sets via the SALUS Interoperability Framework.

This section depicts the complete transformation and mediation cycle of the query and the results, which is initiated by the CSCT by passing the query parameters to the Safety Analysis Query Manager (SAQM). SAQM is responsible for forwarding the eligibility criteria represented in SALUS CIM Ontology to the registered data sources and getting back the aggregated results again in SALUS CIM. The complete cycle is presented in detail in Figure 5. SIL-DS components at each site localize the query in SALUS CIM to HQMF in Lombardy and SPARQL query compliant with ORBIS Content Entity Model in TUD. After query execution, result sets are first converted to local models and then to SALUS CIM Ontology instances. Merging is handled by the Aggregation Service.

Now, all the patient data in SAQM are represented in SALUS CIM; however, yet it is not possible to “understand” as they are coded with several codes from different terminology systems.

### 3.6. Terminology Reasoning

The first step to overcome the terminology reasoning challenge is the representation of the terminology systems as ontologies within the SALUS Semantic Resource Set. For this, we prefer the well-established Simple Knowledge Organization System (SKOS) [74] vocabulary. We create a skos:Concept for each code in the terminology system and define the skos:inScheme property to semantically link the concept (i.e., code) to the encapsulating concept scheme (i.e., the terminology system). Each concept is identified with URIs, which are persistent and hence easily discoverable through the Linked Open Data principles [75]. We adapted MedDRA, SNOMED-CT Clinical Findings subhierarchy, ICD-9-CM, ICD-10, WHO-ATC, and HL7 AdministrativeGender from BioPortal [76]. When a terminology system is not available in BioPortal, we create its semantic representation ourselves, as in the case of ICD-10-GM (German Modification).

The next step is formalizing the mapping between terminology systems. We utilize several reliable terminology mapping resources for this purpose, as presented in Table 1.

In order to realize terminology reasoning at run time in acceptable durations, it is absolutely necessary to do some in advance inferencing specific to the reasoning requirements, which is known as materialization in the semantic Web domain.

**Figure 6:**
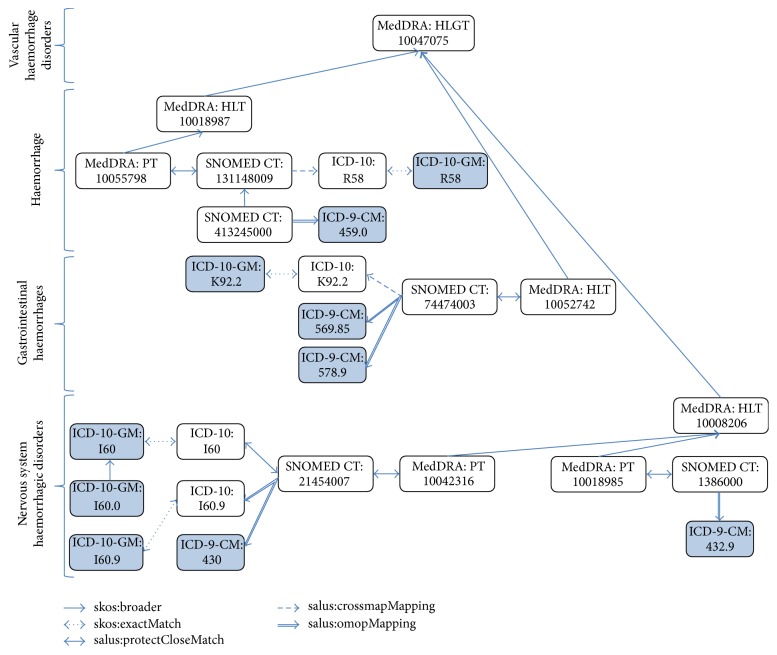
Some generic and specific codes for representing* Haemorrhage* and the relations among them, as they are available in the SALUS Semantic Resource Set.

In our case series characterization scenario, the conditions of the patients are provided with several codes at different levels from ICD-9-CM in Lombardy and ICD-10-GM in TUD. However, the safety analyst wants the conditions to be grouped under a different terminology system, namely, MedDRA, and also at a specific level in the MedDRA hierarchy, in this case HLGT. Therefore, we should be able to find either exact or broad correspondences of various source codes from ICD-9-CM and ICD-10-GM to MedDRA HLGT terms. An example for* Haemorrhage* is presented in Figure 6. The actual codes used in the source EHRs are shaded in this figure. We are expected to group all these codes under the MedDRA HLGT code “10047075” for* vascular haemorrhagic disorders*, although it does not have a direct link with any of these (shaded) codes used in source EHRs.

In our Semantic Resource Set, we represent the original hierarchical relationships within a terminology system with “skos:broader” property. Regarding the mapping across terminology systems, we have used a number of resources providing the mapping across different terminology systems and formally represented them through RDF properties.

- IMI PROTECT project created an ontology called OntoADR, which also presented the correspondence between MedDRA and SNOMED-CT codes [73]. We represented the mapping provided by the OntoADR ontology between SNOMED-CT and MedDRA codes through the “salus:protectCloseMatch” property.
- OMOP project [27] provides mapping of a selected subset of ICD-9-CM and ICD-10-CM codes to SNOMED-CT Clinical Findings. OMOP project has a similar objective with SALUS project, which is to map the ICD codes used to code clinical conditions in EHR sources to SNOMED-CT codes, as SNOMED-CT codes are used as pivot terminologies through which statistical analysis is carried out. We represented the mapping provided by OMOP project between SNOMED-CT and ICD-9-CM codes through the “salus:omopMapping” property. The mapping provided for ICD-10-CM was mostly covering the leaf nodes of ICD-10-CM and missing almost all intermediary nodes. Hence, we have utilized another resource for these mapping.
- US NLM provides mapping between SNOMED-CT and ICD-10 to support semiautomated generation of ICD-10 codes from clinical data encoded in SNOMED-CT for reimbursement and statistical purposes. This is a result of CrossMap Project by IHTDSO and WHO [77]. The original CrossMap mapping is expressed in spreadsheets, where SNOMED-CT codes are mapped to ICD-10 codes with additional context information represented through custom rules. We have represented this mapping in our Semantic Resource Set through the “salus:crossmapMapping” property.

It should be noted that, in our first attempt, we tried to represent this mapping through the well-established SKOS ontology via its relationships like skos:exactMatch and skos:narrowMatch and used these relationships to infer mapping between ICD-10-GM and MedDRA. However, after manually analysing some of the inferred terminology mapping, we realized that there is clinically incorrect mapping. We discovered that most of the errors are due to the transitive and bidirectional nature of SKOS mapping relationships [78]. After some inferencing, the mapping may bring assertions that a mapping creator (such as OMOP, Protect, or CrossMap) may not have intended. Furthermore, those assertions may also conflict with existing semantic or mapping relations. For these reasons, we have created specific mapping relationships, such as salus:omopMapping, salus:protectCloseMatch and salus:crossmapMapping.

**Figure 7:**
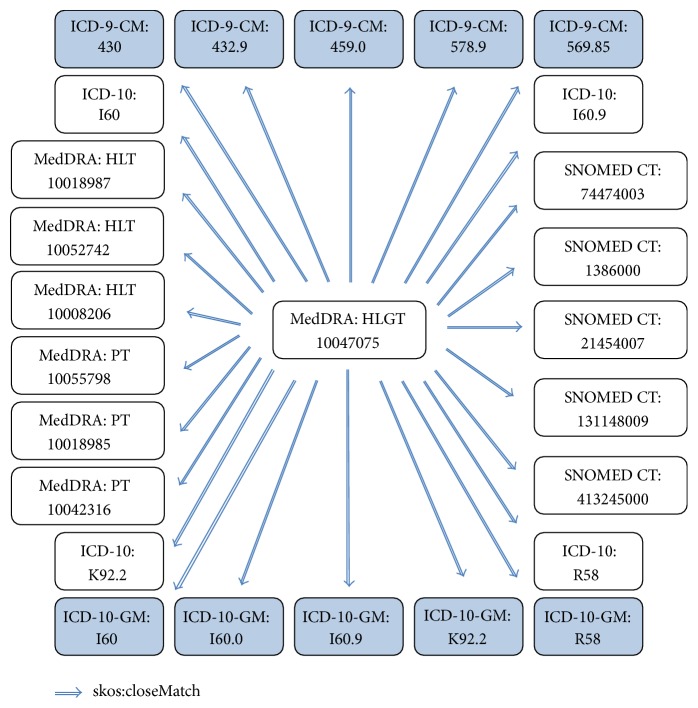
An excerpt from the result of “skos:closeMatch” relationship transitive closure calculation.

By using all these relationships, in this scenario we apply a series of terminology reasoning rules, again implemented on top of EYE, which calculate the full transitive closure of “salus:closeMatch” relationship for all the codes in our Semantic Resource Set. A part of the result for the haemorrhage example is provided in Figure 7. As displayed in the figure, now it is possible to reach the broad MedDRA HLGT “vascular haemorrhagic disorders” term with a single link, not just for ICD-9-CM or ICD-10-GM codes used in the source EHRs, but also for all the relevant codes involved in the materialization process from other systems such as SNOMED-CT.

These materialized results are provided to the Terminology Reasoning Service. At run time, Terminology Reasoning Service is used to enrich the coded information in retrieved population data, such as problem and active ingredient, with the codes from the terminology systems preferred by the safety analyst. This materialized mapping information is also used while querying the EHRs, for query expansion. In Lombardy DWH, for example, the original query for* Pancreatitis* MedDRA code is expanded with all corresponding identical codes in ICD-9-CM and their children if present, because the Lombardy DWH is unaware of MedDRA.

### 3.7. Query Result Calculation

**Figure 8:**
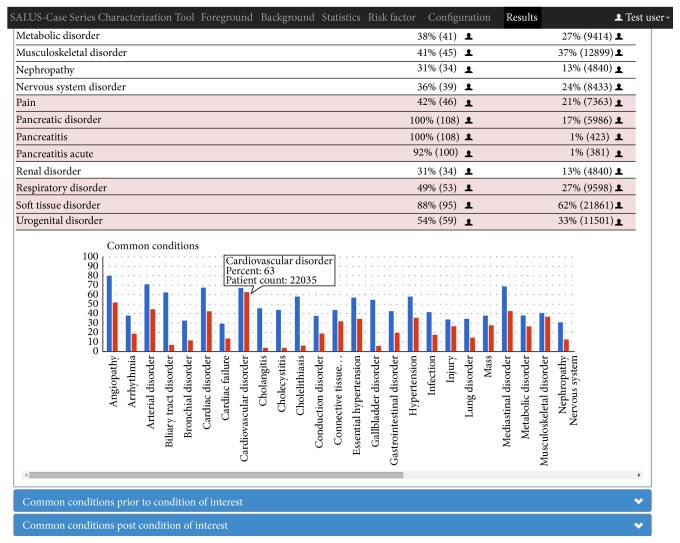
A part of overall common conditions results displayed by the CSCT.

The final step is the calculation of the statistics that the analyst asked for. Queries implemented as EYE rules are executed on the patient data enriched as a result of terminology reasoning to extract the common and different characteristics of the foreground and background populations. The results are displayed by the CSCT, as seen in Figure 8. Each information box corresponds to a statistics configuration (age, gender, country of origin, overall common conditions and medications, and common medications/conditions before/after medication/event of interest) that the safety analyst did during the query. Each box first presents the name of an item and then its occurrence rate and number of occurrences within foreground and background populations together with a graphical chart view.

When the* Ramipril* and* Pancreatitis* example is executed on the Lombardy DWH containing 1 million patients, 34773 patients are found as the background population (those taking* Ramipril*) and 108 patients are found as the foreground population (those having* Pancreatitis* within 120 days of* Ramipril* intake), which accounts for 0.31% of the background population. When the foreground population is defined without the temporal relation, that is, all patients who have* Ramipril* and* Pancreatitis* in their medical records, 423 patients that account for 1.22% of the background population are found.

Upon the previous configuration of the analyst, all the conditions of the background and foreground populations are grouped under MedDRA HLGT terms and presented comparatively. Similarly, the medications are grouped by their active ingredients at the substance level. By analysing all these results, the safety analyst decides in an informed manner whether a specific drug (*Ramipril* in this case) can be attributed as the major cause of an event (*Pancreatitis* in this case), or there are other reasons more likely to cause the event than the exposure drug, such as age and comorbidities (e.g.,* Diabetes*) or other drugs. It is also possible to see the details of a single patient in an anonymized manner through triggering the SALUS Patient History Tool within CSCT, by clicking the patient icons in each information item. For each patient, the analyst can inspect and analyse the patient summary with all the information related to hospitalization, ambulatory events, allergies, drugs intake, and vaccinations.

The quantity and quality of the information provided by SALUS CSCT to the UMC safety analysts are a significant improvement compared to what they are able to access using traditional methods based on reported ADEs and without access to EHR sources.

## 4. Results and Discussion

CSCT and all related components have been implemented and deployed on top of the SALUS Semantic Interoperability Framework integrated with the central Data Warehouse (DWH) of the Lombardy Region. This regional DWH contains anonymized structured data of about 16 million patients with over 10-year longitudinal data on average. There are around 1 billion medical records grouped as follows:

- ~550 million ambulatory diagnoses;
- ~275 million drug prescriptions;
- ~80 million conditions;
- ~35 million vaccinations;
- ~30 million inpatient diagnoses;
- ~2 million allergies;
- ~800.000 pregnancy records.

We have followed a progressive deployment approach to effectively address challenges due to technical integration and testing with huge data and started with deploying incrementally on 3 reduced subsets of the original DWH including 40, 100 thousand, and 1 million patients. After ensuring stability and optimum parameters for parallel execution of subqueries to improve the performance, we have deployed on the DWH with 16 million patients.

All deployment activities have taken place within the care zone of the data owners, and remote validators (i.e., pharmacovigilance researchers) in the research zone accessed the SALUS safety analysis tools including CSCT, which are all implemented as Web applications, through secure VPN channels and access credentials. There is no transfer of identified patient data outside the care zone; only anonymized data are accessible. The deidentification process has been carefully built and put in place. All personal information has been anonymized; date of birth has been generalized; date of death and event dates have been randomly and coherently shifted; rare diseases and orphan drugs have been eliminated.

The validation activities for the Lombardy pilot application took place from August 2014 to January 2015 for all SALUS tools with the involvement of several experts from UMC and Lombardy. These activities and results are presented in the following subsections.

### 4.1. End-User Validation

In order to assess whether CSCT fulfills the intended use from an end-user point of view, it has been tested and evaluated by real end-users from UMC and Lombardy Regional Pharmacovigilance Centre in the scope of the SALUS project. The SALUS Evaluation and Validation Framework has been developed based on the ISO/IEC 25040 Systems and software engineering - - Systems and software Quality Requirements and Evaluation (SQuaRE) - - Evaluation process. According to the developed framework, 4 pharmacovigilance researchers (3 research pharmacists with 2–4 years and 1 senior researcher with more than 15 years of experience in pharmacovigilance and signal detection) from UMC and 2 pharmacovigilance researchers (statisticians with 10 years of experience) from the Lombardy Regional Pharmacovigilance Centre have taken part in the evaluation in order to assess the feasibility of conducting a case series characterization study over the huge Lombardy DWH by using CSCT. These 6 pharmacovigilance researchers have tested the CSCT with hundreds of different query combinations from real-life.

A few queries with their durations of execution on two different DWHs of Lombardy, that is, with 1 million patients and with 16 million patients, are provided in Table 2. In these sample queries, the foreground and background populations are defined similarly to the demonstrated query in the previous section, that is, medication + reaction within 120 days as the foreground and medication only as the background patient population. As the defined population becomes more common and the number of eligible patients increase, the execution time of CSCT increases as well.

In line with our ISO/IEC SQuaRE compliant evaluation and validation framework, in order to collect and analyse end-user feedback, we have developed online questionnaires addressing different validation characteristics including* usability*,* efficacy*,* viability,* and* social acceptance* by utilizing standards based scales, namely, System Usability Scale (SUS) and Health IT Usability Evaluation Scale (Health-ITUES). The scores obtained from the questionnaire based evaluation for CSCT range from average to above average, as shown in Table 3. Considering that these averages are not for prototypes but for real products, we can conclude that the end-users are satisfied and confident with the CSCT.

In the questionnaires, the end-users have agreed on the following aspects.

- CSCT is an added value to the existing process of research in pharmacovigilance.
- CSCT makes it easier to define eligibility queries and retrieve eligible patients for foreground and background populations.
- CSCT is compliant with the existing local, regional, and national processes.

We have also carried out focus group meetings and interviews with the validator end-users. The most prominent positive comments of the CSCT regarded its general user friendliness and ease of use. An average time of 7–10 minutes was required in order to get acquainted with the tools before team members felt confident in how to use them. Other positive aspects that were mentioned included the possibility of selecting different credibility intervals in certain statistical measures and more generally that the tool indeed has the potential to provide useful information in signal detection and validation work.

The major criticism of the CSCT regarded the time it takes to execute the queries, especially when the eligible patient population retrieved as the result of a query is big. This is due to the huge amount of patient records being accessed remotely in real-time and heavy use of standards based transactions, semantic conversion, and terminology reasoning operations, which the end-users have accepted as well. This criticism came from UMC experts, who are used to working on top of locally stored data which is converted in advance to formats and terminology systems used in the clinical research domain and hence not subject to several conversions for interoperability, such as the studies done on central data repository of the OMOP initiative [27] (by the way, in SALUS we have also developed interoperability solutions to populate an OMOP data repository automatically from data in the EHR systems, but these are used in other pilot application scenarios and hence not within the scope of this paper). Yet, one researcher from UMC recommended that it would be good to get some sort of time bar, indicating the remaining time to when the query is expected to be completed. There were further suggestions for improvement; for example, although the CSCT has a user guide document, one researcher recommended that it would be good to have more information boxes/instructions on the different pages in the CSCT, especially in the configuration page.

Further details on end-user validation of CSCT and all other SALUS ADE detection and safety study tools are presented in SALUS D7.2.2 Validation Report for SALUS Pilot Application [79].

### 4.2. Comparative Analysis

Lombardy Region is planning for a drugs monitoring project for adverse reactions specifically for patients treated with new oral anticoagulants (NOACs). Before initiating this project, Lombardy Regional Pharmacovigilance Centre carried out a preanalysis study with the available data in the Lombardy DWH to investigate the relationships between NOACs and some medical conditions as suspected ADEs (e.g., dabigatran etexilate as the NOAC and upper gastrointestinal haemorrhage as the suspected ADE), by using traditional methods and tools supported with custom-built queries and manual interpretation of data. After deploying SALUS tools on top of the Lombardy DWH, experts from Lombardy Informatics (LISPA; the partner in the SALUS project from Lombardy) decided to repeat the same study by using the CSCT, which provided the opportunity to test CSCT and the underlying SALUS Semantic Interoperability Framework in the field.

This comparative analysis revealed that the results provided by CSCT were identical with those found by the Lombardy Regional Pharmacovigilance Centre through traditional methods, which confirmed the technical correctness of our implementation. The main difference was observed in terms of time and resources spent to complete the studies. Experts at the Lombardy Regional Pharmacovigilance Centre reported that they completed their NOAC study in 1 month using traditional methods, while it took only 2 full days to repeat the same study by using CSCT and the underlying interoperability platform. Experts from the Lombardy Regional Pharmacovigilance Centre were impressed with this significant improvement of time and resource utilization.

### 4.3. Discussion

The adoption of EHR systems and data exchange among these systems are rapidly increasing due to a number of national and cross-border projects in Europe and Meaningful Use in the US [80]. A majority of these initiatives employ well-accepted content and transaction standards/profiles such as CDA, CCD, and IHE X^*∗*^ [81]. For example, in Turkey, episodic medical records of the whole population (~75 million) are collected from the healthcare providers as CDA documents since January 2009 [82]. Thanks to epSOS, which was a large-scale European pilot for exchange of electronic patient summary and prescription documents across borders, many European national infrastructures are now able to provide and consume patient data in PCC/CCD templates [83]. Hence, both institutional and regional/national EHR systems become more and more standards compliant.

Although the main priority of these systems is improving clinical care, we demonstrate that the same systems and interfaces can be exploited for postmarket safety studies as well, with minimum intrusion when necessary, as in the case of our QED extension for population based queries. Our implementation proves that it is possible to carry these observational studies without developing study specific databases and Data Warehouses, which is costly and hard to maintain.

In the TUD case, we also demonstrate a complementary approach by developing a semantic interface directly on top of the EHR database and formalizing patient data immediately. This approach is of course more capable in the sense that the whole content of the EHR database can be formalized and more complex querying can be done compared to the standard based interfaces for data exchange. However, it necessitates an in-depth knowledge of and interaction with the storage structure of the EHR system, in addition to expertise with semantic Web technologies. Our advantage in SALUS is that AGFA as the developer of the ORBIS system is a core beneficiary of the project, so that we are able to demonstrate both approaches in parallel in integrated scenarios.

One of the biggest challenges in developing semantic Web applications is utilizing a satisfactory reasoning engine that is able to perform in reasonable time and space. In our very early prototype [51], we were able to overcome this challenge by limiting our reasoning requirements to the minimum and meeting those with Virtuoso triple store [84]. However, we had more complex reasoning requirements in the actual pilots with real data, which we resolved by using EYE Reasoning Engine in semantic processing and reasoning operations in the SALUS architecture. The best thing about EYE is that you get what you ask for, nothing more, nothing less.

The data that we need in SALUS scenarios such as conditions, procedures, allergies, and medications of the patients are always available in a structured manner in the Lombardy DWH. On the other hand, we have observed in TUD that some medical details of some patients are only available in free-text patient documents and are missing in a structured manner. This naturally limits the benefits of our advanced safety study tools. However, analysis of free-text data in EHRs was not within the scope of the SALUS as a focused research project.

Last but not least, it is very critical to have reliable and explicit mapping between terminology systems to accurately address the Semantic Interoperability challenge between clinical care and clinical research domains. In SALUS, we have analysed several mapping resources and represented the best options in RDF through SALUS specific properties mostly, and, through reasoning, we have inferred close matches that can be of use to SALUS end-users. It was not always possible to infer stronger and more valuable relationships such as exact match due to missing semantics. Therefore, in order to make the existing mapping reliable and reusable over the semantic Web, it is extremely important that the communities, who create the mapping, provide them in RDF using standard ontologies such as SKOS to indicate the exact semantics of the mapping relationships.

## 5. Conclusions

We have developed a scalable interoperability framework for observational studies and demonstrated in this paper how it is used for case series characterization by the pharmacovigilance researchers. Through our integration, validation, and comparative analysis studies, we have proven that the CSCT and the underlying SALUS Semantic Interoperability Framework have gone beyond simple proof-of-concept prototypes.

Semantically mediating all the patient data and terminology systems in formalized representations allows us to extend the capabilities of our tools via introduction of new rules easily. For example, we are able to insert a new rule to check the existence of diabetes through age, some specific medications (e.g., metformin), and laboratory test results (e.g., glycosylated hemoglobin) when diabetes is not explicitly recorded in the list of diagnoses of a patient.

Scalability is due to our semantic mediation approach; whenever a new source or target content model is to be added, the required mapping to the SALUS CIM is added in linear time, without affecting the existing resources. For example, although not used directly in our pilot sites, recently we have also added ISO/CEN 13606 archetypes as another source model. Furthermore, our decoupled RESTful services allow us to improve the overall performance by multiplying the services for concurrent processing and reasoning.

The SALUS architecture is designed for all kinds of observational studies, not just for case series characterization. In our other pilot application scenarios (e.g., temporal pattern characterization for signal detection), we have additional requirements such as subscribing to population data and mapping population data to OMOP CDM as the target model. We have implemented the necessary supplementary components for meeting these requirements and validated the involvement of several end-users as in the case of CSCT.

As one of the final outcomes of the SALUS project, we have developed a guidance document [85] targeted to anybody involved in defining and/or implementing strategies for increased availability, use, and quality of EHR content for postmarketing drug safety studies. In this document, we have clearly described the building stones of SALUS and the supporting ADE detection and reporting and safety analysis tools and provided a roadmap to adopt advanced ADE reporting and postmarket safety study mechanisms by taking into account the different levels of maturity in the interested regions/countries and by explaining the necessary preparation, installation, testing, validation phases with clarity. The document is supported by the SALUS Starter-kit [86] that includes all the developed software as open source components, corresponding documentation, and screencasts.

Beyond the project, SALUS partners are now concentrating on the exploitation and marketing of the SALUS Semantic Interoperability Framework and the supporting ADE detection and safety analysis tools. The most concrete efforts are taking place in the pharmacovigilance authorities in Lombardy, Italy, and in Turkey for large-scale deployment and operational use at the regional and national levels.

## Figures and Tables

**Table 1:** Terminology mapping resources that are utilized in the SALUS Framework.

Source system	Target system	Number of mappings	Mapping resource
MedDRA	SNOMED-CT	10,648	OntoADR of the PROTECT project, manual improvement of UMLS mapping by PROTECT experts [73]
ICD-9-CM	SNOMED-CT	16,819	OMOP Vocabulary, created manually by experts
ICD-10-CM	SNOMED-CT	59,122	OMOP Vocabulary, created manually by experts
SNOMED-CT	ICD-10	27,166	CrossMap, a collaborative project by IHTSDO and WHO
ICD-10-GM	ICD-10	12,318	Identical codes in both systems
ICD-9-CM	SNOMED-CT	43,086	BioPortal, manual review by SALUS experts before inclusion
ICD-10-CM	SNOMED-CT	45,022	BioPortal, manual review by SALUS experts before inclusion

**Table 2:** A few CSCT queries and their execution times on two Lombardy DWHs with 1 million and 16 million patients.

Medication	Reaction	Execution time in 1 million patients	Execution time in 16 million patients
Dabigatran	Upper gastrointestinal haemorrhage	40 minutes	0.4 days
Nifedipine	Acute myocardial infarction	95 minutes	1.6 days
Simvastatin	Rhabdomyolysis	543 minutes	6 days
Ramipril	Pancreatitis	647 minutes	7.2 days

**Table 3:** CSCT questionnaire based evaluation scores (italic: average; bold: above average; the interval is [1–100] for usability score and [1–5] for the rest).

Usability	Social acceptance and viability	Quality of work life	Perceived usefulness	Perceived ease of use	User control
*64*	*3.1*	*2.7*	*3.3*	**3.9**	*2.7*

## Abbreviations

ADE: * Adverse Drug Event*
BRIDG: * Biomedical Research Integrated Domain Group*; it develops a domain analysis model that aims to produce a shared view of the dynamic and static semantics for protocol-driven research and its associated regulatory artifacts
CDE: * Common Data Element*, the smallest meaningful data container in a given context
CDISC: * The Clinical Data Interchange Standards Consortium, *a global, open, multidisciplinary, nonprofit organization that is establishing standards to support the acquisition, exchange, submission and archival of clinical research data and metadata
CDISC CDASH: * CDISC Clinical Data Acquisition Standards Harmonization*; it describes recommended basic standards for the collection of clinical trial data
CDISC ODM: * CDISC Operational Data Model*, a standard that facilitates the archive and interchange of the metadata and data for clinical research
CEN: * The European Committee for Standardization*
CIM: * Common Information Model*; the model id composed of CDEs for representing common knowledge in a given context and acting as the mediator among different models
CSCT: * Case Series Characterization Tool*
DWH: * Data Warehouse*
EHR: * Electronic Health Record*
EYE: * Euler Yap Engine*, an open source and high performance reasoning engine
HITSP: * Health Information Technology Standards Panel*, a public-private partnership harmonizing and integrating standards that will meet clinical and business needs for sharing information among organizations and systems
HL7: * Health Level Seven*, a nonprofit organization involved in the development of international healthcare informatics interoperability standards
HL7 CDA: * HL7 Clinical Document Architecture*, a document markup standard that specifies the structure and semantics of a clinical document for the purpose of exchange
HL7 HQMF: * HL7 Health Quality Measures Format*, a standard for representing a health quality measure as an electronic document, which also allows for defining inclusion/exclusion criteria for a specific patient population
HL7 RIM: * HL7 Reference Information Model*, the shared model among all HL7 domains and, as such, is the model from which all domains create their messages and documents
HL7/ASTM CCD: * HL7/ASTM Continuity of Care Document*; it defines a number of constraints on HL7 CDA standard to foster interoperability of data about a patient among health professionals without loss of meaning
ICSR: * Individual Case Safety Report*; it captures information about Adverse Drug Events that are reported to public health organizations or regulatory bodies
IHE: * Integrating the Healthcare Enterprise*, a nonprofit “integration organization” promoting the coordinated use of established standards to address specific clinical need in support of optimal patient care
IHE PCC: * IHE Patient Care Coordination*, an IHE domain dealing with general clinical care aspects such as document exchange and order processing. It further details and multiplies the HL7/ASTM CCD templates at the document, section and clinical statement levels
ISO: * International Organization for Standardization*
N3: * Notation 3*, an assertion and logic language which is a superset of RDF. It also provides a textual syntax alternative to RDF/XML
OMOP: *Observational Medical Outcomes Partnership*, a public-private partnership trying to identify the most reliable methods for analysing huge volumes of clinical data drawn from heterogeneous sources. OMOP develops the Common Data Model in order to standardize the data format used in disparate data sources for the purposes of clinical research
OWL: * Web Ontology Language*, a set of knowledge representation languages maintained by World Wide Web Consortium (W3C) for authoring ontologies
RDF: * Resource Description Framework*, a set of specifications maintained by the World Wide Web Consortium (W3C) for conceptual modeling of data (describing meta-models) through a variety of syntax notations and data serialization formats
REST: * Representational State Transfer*, a software architecture style which was developed by W3C for designing distributed systems
SALUS: * Scalable, Standard based Interoperability Framework for Sustainable Proactive Post Market Safety Studies*, European Commission supported research project addressing the interoperability issues between clinical research and patient care domains for pharmacovigilance activities
SAQM: * Safety Analysis Query Manager*
SIL: * Semantic Interoperability Layer*
SKOS: * Simple Knowledge Organization System*; it provides a model for expressing the basic structure and content of concept schemes such as thesauri, classification schemes and taxonomies as well as other similar types of controlled vocabulary
SPARQL: * SPARQL Protocol and RDF Query Language*, a query language for retrieval and manipulation RDF data
TUD: * Technical University of Dresden*
UMC: * WHO Collaborating Centre for International Drug Monitoring, Uppsala Monitoring Centre*
UMLS: * Unified Medical Language System*, a compendium of key terminology, classification, and coding standards in the biomedical sciences
WHO: * World Health Organization*
XML: * Extensible Markup Language*
XSD: * XML Schema Definition*
XSLT: * Extensible Stylesheet Language Transformations*, a language for transforming XML documents into other XML documents or objects.
